# Income gradient in health-related quality of life — the role of social networking time

**DOI:** 10.1186/s12939-019-0942-1

**Published:** 2019-03-15

**Authors:** Shaozhe Zhang, Wei Xiang

**Affiliations:** 1 Department of Sociology, Wuhan University, Wuhan, 430072 China; 2 Office of Medical Affairs, Union Hospital, Tongji Medical College, Huazhong University of Science and Technology, Wuhan, 430022 China

**Keywords:** Health-related quality of life, Income, Gradient, Social networking time

## Abstract

**Background:**

Widening social class discrepancies in health persist in the United States. Although the relationship between social class and health has been well illustrated, the pathways through which social class influences the distribution of health remain unidentified. This study is designed to analyze the income-health relationship by examining the role of social networking time.

**Methods:**

A nationwide sample from the General Social Survey of the United States is adopted for the statistical analysis. The Healthy Days Measures developed by the Centers for Disease Control and Prevention are used to evaluate health-related quality of life in the general population. Social networking time is measured through the number of social evenings respondents spend with neighbors. Individuals’ inflation-adjusted family income is used to indicate their income. The relationships between income, social networking time and health-related quality of life are calculated through multiple linear regressions, and the mediation effects of social networking time are further tested by the Sobel test with bootstrapping.

**Results:**

People with a lower income tend to spend more time socializing with their neighbors than those with a higher income. Income is positively associated with health-related quality of life. Respondents who engage more frequently in neighborhood socializing report poorer health-related quality of life. The reproduction of the income gradient in health-related quality of life through social networking time mainly persists in mental health aspects.

**Conclusions:**

This study verifies the positive association between income and health-related quality of life. The results show that people’s network ties are affected by their income and confirm the role of social networking time in the reproduction of the income gradient in health-related quality of life.

## Introduction

In recent decades, life expectancy has increased and the mortality rate has decreased, but socioeconomic disparities in health outcomes persist. Social gradients in health, whereby individuals from lower social class backgrounds have poorer health status, higher risk of illness and a shorter life expectancy, have been well documented in existing studies [1–4]. Numerous efforts, such as increased investment in health and health services and the implementation of health promotion policies, have been made to reduce health inequalities; however, substantial evidence indicates that social class disparities in health are widening [5–8]. Income, as a basic indicator of social class, plays a dominant role in the maintenance of people’s health [9]. Higher income is related to better health conditions and lower health risks, while lower income means more exposure to health risk factors. How is income related to health, and why is the income gradient in health continuing to widen?

Prior studies on the income-health relationship focus on two sets of issues: the effect of absolute income on health and the association between relative income and health. With regard to the effect of absolute income, money involves access to resources that benefit for health, such as medical services [10], better nutrition [11] and resources embedded in social networks [12]. The relative income aspect mainly concerns the psychosocial pathways of the income gradient in health, as the sense of relative deprivation that derives from income differences could have adverse psychosocial consequences [13]. People with lower relative income experience higher risks of mental health disorders, including depression and anxiety [14].

Among all the factors linking income and health, the effect of social networks attracts the most attention. However, the measurements of social networks vary across studies, and social networks are often conceptualized in terms of both functions and structures. The functional conceptualizations include supportive social contacts [15] and the availability of support [16]. As a main source of social support, social networks are considered protective factors of health. The structural aspects of social networks encompass the social ties in which people are embedded [17] and the pathways of network influence [18]. Smith and Christakis [19] reviewed the differences between social support and social networks and found that studies on social support focus on the number of contacts and the helpfulness of those contacts, while analyses of social networks concentrate on the nature and types of ties linking people together, including the closeness of the ties and the relationships of the people embedded in the ties, such as friends, relatives and neighbors. The influence of social networks on health appears uncertain when networks are conceptualized structurally. Different types of social ties are not always supportive of or beneficial for health, as a negative effect of neighborhood connections on mental health has been found [20].

Moreover, although income is positively related to the availability of social support, people of different income levels have distinctive preferences in relation to social network types. A study on the association between income and social network choices shows that people with higher income spend more time with their friends, while those with lower income spend more social networking time with neighbors [21]. Since income impacts people’s social networking time with their neighbors and neighborhood connections might be harmful for health, is it possible that neighborhood networks mediate the relationship between income and health and help explain the health inequalities between people with different income levels?

This study extends the current research on the income gradient in health by discussing the possible mediating effect of a typical type of social network: neighborhood ties. Income is considered a social contextual factor influencing people’s embeddedness in neighborhood ties. Furthermore, health-related quality of life (HRQoL) is adopted to evaluate people’s health outcomes [22–24]. HRQoL is described as people’s perceived physical and mental health over time [25], reflecting individuals’ subjective feelings of the extent to which health problems influence their daily life. In the context of the epidemiological transition to an era in which non-communicable chronic diseases have become a major risk factor for people’s health, HRQoL, which implies a direct linkage to health conditions [26] and subjective assessment of health status, is more reflective of the modern biopsychosocial medical model than objective indicators such as life expectancy and mortality rate [24].

## Methods

### Data

The cross-sectional data employed for the statistical analysis were drawn from the General Social Survey (GSS) 2002, 2006, 2010 and 2014 [27]. Launched in 1972, the GSS gathers data on contemporary American society to monitor and explain trends and constants in the attitudes, behaviors, and attributes of the adult population in the United States. With the support of the National Science Foundation, the GSS is conducted by the National Opinion Research Center (NORC) at the University of Chicago every one or two years with a strict, full-probability sample design, and the response rates range from 70 to 82.4% [28]. In each survey year, subsamples are randomly selected to answer selected survey questions. HRQoL questions were asked in 2002, 2006, 2010 and 2014, and participants in the subsamples who answered the questions in these four survey years were included in the analysis.

### Variables

#### Dependent variables

The dependent variable, HRQoL, was assessed by the “Healthy Days Measures” developed by the Centers for Disease Control and Prevention (CDC) [29]. These measures include four questions on general health, the number of physically unhealthy days, the number of mentally unhealthy days, and the number of days with activity limitations. General health is assessed with the question “Would you say that in general your health is excellent, very good, good, fair or poor?” Responses range from 5 to 1. The number of days of poor physical health, poor mental health and activity limitations are assessed, respectively, with the questions “Now, thinking about your physical health, which includes physical illness and injury, how many days during the past 30 days was your physical health not good?” “Now, thinking about your mental health, which includes stress, depression, and problems with emotions, how many days during the past 30 days was your mental health not good?” and “During the past 30 days, approximately how many days did poor physical or mental health keep you from doing your usual activities, such as self-care, work, or recreation?”. The validity of the Healthy Days Measures for measuring HRQoL was confirmed by comparing their outcomes with those of the SF-36 in two previous studies using special samples and statewide samples in the US [30, 31]. The reliability of the Healthy Days Measures was also shown to be excellent in a retest study carried out in the US [32].

#### Independent variable

The independent variable in this analysis was income. The family real income variable (REALINC) in the GSS data, which is inflation-adjusted constant dollars [33], was used in the analysis. Family income was used rather than the respondent’s income because in the GSS, family income measures income from all sources, while the respondent’s income is the earnings from a single occupation [33]. The income variable was log transformed in the models.

#### Mediation variable

Social networking time served as the possible mediator in illustrating the reproductive path of income gradients in HRQoL, and it was represented by the number of social evenings the respondents spent with their neighbors per year. This was evaluated with the question “How often do you spend a social evening with someone who lives in your neighborhood?” in the GSS. The original categorical frequencies were transferred into numeric days per year by assigning “almost daily” a value of 300, “once a year” a value of 1 and “never” a value of 0. A value of 4 was assigned to the “several” response. Thus, “several times a week” was coded as 208 (4 × 52), “several times a month” as 48 (4 × 12) and several times year as 4 (4 × 1) [21, 34].

#### Control variables

Sociodemographic variables that have been confirmed to affect HRQoL were employed as control variables, including age, sex, race, marital status, place of living and working status. Number of children and geographical mobility (whether the respondent had lived in the same city since age 16) were further controlled to assess the robustness of the relationship between income and social networking time with neighbors. Dummy variables for the GSS survey year were also included because the size of people’s social networks declines over time [35].

### Analytical strategies

**Fig. 1:**
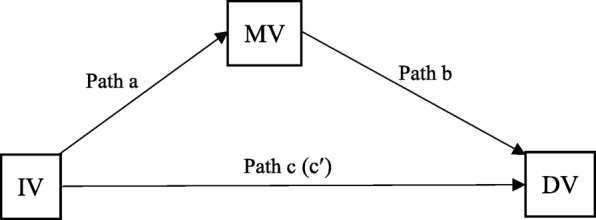
Mediation effect paths

The objectives of this research were twofold. First, the study examined the relationships between income, social networking time and HRQoL. Then, the reproduction of health inequalities through social networking time was tested based on the four criteria for mediation paths proposed by Baron and Kenny [36] (Fig. 1): (1) the coefficient of path a is significant in identifying the effect of the independent variable (IV) on the mediating variable (MV); (2) the MV is significantly related to the dependent variable (DV) net of the IV (path b); (3) the significant direct association (path c) between the IV and the DV is confirmed; and (4) the association between the IV and the DV is weakened when the MV is controlled (path c′).

Stata/MP 14.2 for Mac was used to carry out the statistical analysis. Multiple linear regression models were formulated to evaluate the associations among income, social networking time and HRQoL. First, the way that individuals’ income predicts social networking time with neighbors was tested. Two models were constructed for this question. Sociodemographic control variables were included in Model 1, and variables of geographic mobility and number of children were added to Model 2 based on Model 1. Then, the relationship between income and HRQoL was calculated, and social networking time was further incorporated into the models. Eight models were formulated to assess the relationships. In Models 3, 5, 7 and 9, sociodemographic variables were controlled, and in Models 4, 6, 8 and 10, the indicator of social networking time was included. Third, the possible mediation effect of social networking time was identified using the Sobel mediation test with the bootstrapping process, which overcomes the assumption of a normal distribution of the classical Sobel mediation test [37].

Although social networking time and HRQoL indicators were non-normally distributed, the large sample size (more than 500) justified the use of linear regression [38]. The robustness of the *p* values (Appendix) was confirmed via bootstrapping process [39].

## Results

### Descriptive statistics

The total number of participants with valid responses to the questions on both HRQoL and social networking time in 2002, 2006, 2010 and 2014 was 3330. Table 1 presents the descriptions of all variables in the analysis. The average numbers of days of poor physical and mental health were less than 4 per month, and the number of days of activity limitation was about 1.5 each month. The respondents spent several evenings per month with their neighbors on average.

**Table 1:** Descriptions of the Variables

Variables	Obs	Mean/percent	SD	Value	Value label
Dependent variables					
Days of poor physical health	3322	2.581	6.188	0–30	
Days of poor mental health	3330	3.297	6.964	0–30	
Days of activity limitation	3325	1.230	4.062	0–30	
Assessment of general health	3329	3.652	1.015	1–5	1 = poor, 5 = excellent
Independent variable					
Income ($)	3042	38,281.08	33,832.01	236.5–144,502.7	
Mediator					
Social networking time	3330	54.972	91.971	0–300	
Control variables					
Age	3320	42.898	13.308	18–88	
Sex (male)	3330	48.02%	–	0,1	1 = male 0 = female
Race (white)	3330	75.56%	–	0,1	1 = white 0 = non-white
Marital status (married)	3329	47.10%	–	0,1	1 = married 0 = not married
Working status (working)	3330	97.36%	–	0,1	1 = working, 0 = not working
Geographic mobility (same city since age 16)	3325	39.01%	–	0,1	1 = same city since age 16, 0 = not the same city
Number of children	3327	1.597	1.483	0–8	
Place of living (urban)	3330	89.13%	–	0,1	1 = urban, 0 = rural

Table 2 presents the distributions of the respondents’ social networking time with neighbors, income and HRQoL. Participants who were female, white, married and not working spent fewer social evenings with their neighbors. Female, non-white, and non-married respondents and those living in rural areas had a lower family income. Male and married respondents reported better HRQoL.

**Table 2:** Descriptive results of social networking time, income and HRQoL

Variables	Social networking time		Family income (1000 dollars)		Days of poor mental health		Days of poor physical health		Days of activity limitations		Assessment of general health	
	mean	t-test	mean	t-test	mean	t-test	mean	t-test	mean	t-test	mean	t-test
Sex												
Female	50.269	−9.794^***^	34.672	−7.509^***^	3.704	0.847^***^	2.832	0.521^**^	1.334	0.217	3.639	−0.028
Male	60.063		42.181		2.857		2.311		1.117		3.667	
Race												
Non-white	60.889	7.832^**^	28.077	−13.478^***^	3.131	−0.22	2.425	−0.207	1.157	−0.097	3.557	−0.127^***^
White	53.058		41.555		3.351		2.632		1.254		3.683	
Marital status												
Non-married	69.119	30.007^***^	26.973	−23.821^***^	3.857	1.186^***^	2.796	0.454^**^	1.439	0.443^***^	3.615	−0.078^**^
Married	39.112		50.795		2.671		2.342		0.996		3.693	
Working status												
Not working	38.841	−16.569^*^	37.158	−1.152	8.432	5.274^***^	10.773	8.414^***^	8.432	7.398^***^	3.057	−0.611^***^
Working	55.41		38.310		3.158		2.358		1.034		3.668	
Place of living												
Rural	53.68	−1.453	29.776	−9.568^***^	3.575	0.311	2.756	0.195	1.222	−0.009	3.434	−0.245^***^
Urban	55.13		39.344		3.263		2.56		1.231		3.679	

### Income and social networking time

The association between income and social networking time is displayed in Table 3. In Model 1, a significant negative association was captured between the respondent’s income and the number of social evenings spent with neighbors net of age, sex, marital status, working status and place of living. Geographical mobility and number of children were controlled in Model 2 to check the robustness of the association between income and social networking time. The result indicated that people with a higher income spent less time in their neighborhood socializing than those with a lower income.

**Table 3:** Regression results of the association of income and social networking time^a^

Variables	Model 1	Model 2
Income	−0.133^***^	−0.137^***^
	(1.919)	(1.944)
Age	−0.621^***^	−0.603^***^
	(0.765)	(0.775)
Age^2^	0.534^***^	0.528^***^
	(0.008)	(0.008)
Sex (male)	0.063^***^	0.063^***^
	(3.238)	(3.247)
Race (white)	0.007	0.003
	(3.870)	(3.911)
Marital status (married)	− 0.087^***^	−0.080^***^
	(3.567)	(3.676)
Working status (working)	0.024	0.024
	(10.258)	(10.262)
Place of living (urban)	0.009	0.008
	(5.170)	(5.182)
Geographical mobility (same city since age 16)		0.003
		(3.346)
Number of children		−0.032
		(1.214)
Year fixed effects^b^	yes	yes
*N*	3037	3029
*R* ^2^	0.070	0.071

^a^ Standardized beta coefficients; standard errors in parentheses; ^*^ *p* < 0.05, ^**^ *p* < 0.01, ^***^ *p* < 0.001

^b^ The variables of GSS years were adopted only to control the effect of time, so the statistical parameters are not presented in the table (the same is true in the following tables)

### Income, social networking time and HRQoL

Among the HRQoL measures, income was negatively related to the number of days of poor health and positively related to the general assessment of health (Table 4), reflecting the beneficial role of income in HRQoL. Model 3 and Model 4 calculated how income was related to the respondent’s number of days of poor mental health and the effect of social networking time with neighbors. The result indicated a significant positive association between social networking time and the number of days of poor mental health. Female respondents had more days of poor mental health than male respondents, and white respondents had more days of poor mental health than non-white respondents. In Model 6, respondents who were more engaged in socializing with neighbors had more days of poor physical health, which was similar to the relationship between social networking time and mental health. The effect of the respondent’s social networking time on the number of days of activity limitations and on the general assessment of health was not significant, as shown in Model 8 and Model 10.

**Table 4:** Regression results of income, social networking time and HRQoL^a^

Variables	Days of poor mental health		Days of poor physical health		Days of activity limitations		General assessment of health	
	Model 3	Model 4	Model 5	Model 6	Model 7	Model 8	Model 9	Model 10
Income	−0.127^***^	− 0.118^***^	− 0.106^***^	− 0.102^***^	−0.069^***^	− 0.065^**^	0.219^***^	0.223^***^
	(0.146)	(0.147)	(0.130)	(0.131)	(0.083)	(0.084)	(0.021)	(0.021)
Place of living (urban)	0.002	0.002	0.016	0.016	0.012	0.012	0.049^**^	0.049^**^
	(0.395)	(0.394)	(0.351)	(0.351)	(0.224)	(0.224)	(0.057)	(0.057)
Age	0.082	0.124	−0.010	0.013	−0.043	− 0.026	− 0.443^***^	− 0.424^***^
	(0.058)	(0.059)	(0.052)	(0.052)	(0.033)	(0.033)	(0.008)	(0.009)
Age^2^	−0.141	−0.177	0.066	0.046	0.064	0.050	0.354^**^	0.338^**^
	(0.001)	(0.001)	(0.001)	(0.001)	(0.0004)	(0.0004)	(0.0001)	(0.0001)
Sex (male)	−0.054^**^	− 0.058^**^	− 0.026	− 0.028	− 0.018	− 0.020	−0.012	− 0.014
	(0.247)	(0.247)	(0.220)	(0.220)	(0.140)	(0.141)	(0.036)	(0.036)
Race (white)	0.066^***^	0.065^***^	0.032	0.032	0.031	0.031	0.024	0.024
	(0.295)	(0.295)	(0.263)	(0.262)	(0.168)	(0.168)	(0.043)	(0.043)
Marital status (married)	−0.027	− 0.021	0.005	0.008	−0.024	− 0.022	− 0.031	−0.029
	(0.272)	(0.272)	(0.242)	(0.243)	(0.154)	(0.155)	(0.040)	(0.040)
Working status (working)	−0.116^***^	−0.117^***^	− 0.207^***^	−0.208^***^	− 0.285^***^	−0.285^***^	0.088^***^	0.087^***^
	(0.783)	(0.781)	(0.695)	(0.695)	(0.444)	(0.444)	(0.115)	(0.115)
Social networking time		0.067^***^		0.037^*^		0.026		0.030
		(0.001)		(0.001)		(0.001)		(0.0002)
Year fixed effects	yes	yes	yes	yes	yes	yes	yes	yes
*N*	3037	3037	3030	3030	3032	3032	3036	3036
*R* ^2^	0.045	0.049	0.059	0.061	0.092	0.093	0.063	0.064

^a^ Standardized beta coefficients; standard errors in parentheses; ^*^ *p* < 0.05, ^**^ *p* < 0.01, ^***^ *p* < 0.001

### Mediation effect of social networking time

Model 4 and Model 6 showed that social networking time was significantly related to the respondent’s number of days of poor mental and physical health. Therefore, the mediating effect of social networking time on the impact of income on these two indicators of HRQoL was further tested through the Sobel mediation test with the bootstrapping procedure.

**Fig. 2:**
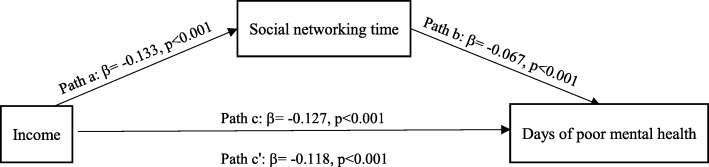
Mediation effect of social networking time on income and mental health

In Model 1, income was significantly related to the respondent’s social networking time with neighbors, which met the requirement of path a (β = − 0.133, *p* < 0.001). In Model 3 and Model 4, the significant association between income and the respondent’s number of days of poor mental health persisted both with and without the adjustment of social networking time. This fulfilled path c (β = − 0.127, *p* < 0.001) and path c′ (β = − 0.118, *p* < 0.001), and the magnitude of income decreased when social networking time was controlled (Fig. 2). The Sobel mediation test indicated a significant indirect effect (Sobel test value = − 0.064, *p* < 0.01), and the result of the bootstrap procedure corroborated the Sobel test result: the 95% bias-corrected CI did not contain zero, indicating that the association of income and the respondent’s mental health was mediated by social networking time.

In Model 5 and Model 6, the coefficients of income and social networking time satisfied the requirement of mediation effect paths; however, the result of the Sobel test was not significant, suggesting that social networking time may not be a mediator between income and people’s physical health.

## Discussion

This study focuses on the income gradient in health and how it is reproduced through people’s social networking time in the United States. A positive association between income and HRQoL is captured, and the inequalities in HRQoL reproduced through neighborhood socializing mainly exist in relation to mental health.

The statistical results demonstrate that people with higher income generally have higher HRQoL, which has been well supported in existing studies [40]. The income gradient in HRQoL derives from various inequalities related to health, including differentiated access to healthcare services and unbalanced allocation of health-related resources, as well as distinctions in health-related lifestyles across social classes.

The study also confirms the negative association between income and social networking time with neighbors. This negative association could be explained by people’s structural embeddedness and their access to resources. Prior analyses show that people with abundant resources tend to be less dependent on others [41, 42]. Thus, people with lower income and limited access to resources may spend more time socializing to obtain social support. A psychological experiment studying patterns of nonverbal displays of disengagement and engagement among participants with varied socioeconomic statuses suggests that people of higher socioeconomic status display more disengagement cues in social contact than people from lower classes [43]. This is in line with the study of Bianchi & Vohs [21], which indicates that people from lower classes tend to spend more time socializing with neighbors than with relatives and friends, as neighbors may provide more immediate instrumental support based on geographic proximity than friends and relatives.

The reproduction of health disparities is examined through the mediating role of social networking time in the relationship between income and HRQoL. The reproduced inequalities are probably more related to mental HRQoL than other aspects, as neighborhood connections impact mental health more directly [44]. Social networks and ties are major sources of social support, which is beneficial for the maintenance of health, but the helpfulness of networks is uncertain. Several existing studies examining mental health inequalities across classes demonstrate that social connections may paradoxically increase the level of mental illness symptoms among less privileged groups [45–47]. From the psychosocial perspective, the sense of relative deprivation arising from social networking with neighbors may harm people with lower income. Reciprocity and mutual instrumental support are the dominant basis of the establishment of neighborhood connections. However, those who are most in need of support are often the least likely to receive it and must bear a disproportionate cost of involvement [48]. People engaging in such relationships with limited resources may face greater difficulty in responding to others’ needs. Therefore, for people with lower income with limited access to resources, reciprocal neighborhood connections mean more psychological costs as a result of the sense of indebtedness. Finally, the contextual characteristics of social networks cannot be ignored in analyzing the reproduction of income gradients in HRQoL through neighborhood ties, as “interpersonal relationships occur within broader social contexts” [49]. People with lower income are more dependent on collective resources in the neighborhood, and neighborhood deprivation may have a negative effect on their mental health. The impact of social context on mental health is indicated by Durkheim in the illustration of the relationship between surrounding social climate and suicide rates [50]. Empirical studies on neighborhood deprivation and mental health indicate that neighborhood attachment is associated with higher reporting of common mental disorders [47]. The sense of relative deprivation, individual neighborhood socializing cost and a comparatively disadvantaged neighborhood environment may jointly or separately result in the reproduction of the income gradient in HRQoL.

This study offers two major contributions. Drawing insights from previous studies on differences in social networks and social support, this study shows that the supportiveness of social networks is uncertain and that social networks are not always helpful and beneficial for people’s health. It further suggests that the social networking time differences between people with different income levels could be considered one of the mechanisms of social class inequality in health.

The study should be viewed in light of several limitations. First, though the significant association between income and social networking time is confirmed, it is uncertain whether neighborhood networking affects people’s income. Although working status is controlled in the analytical models, it is still possible that people who are more engaged in neighborhood socializing are less concentrated on other money-making activities and thus earn less money. Nonetheless, this study maps out people’s social network preferences reconciled with their social positions as a product of their structural embeddedness and surrounding social world. Second, the results indicate that people’s HRQoL is influenced by their social networking time, and the reverse is also possible; that is, people of poorer health status have fewer opportunities to participate in activities other than neighborhood socializing. Finally, income is used as the indicator of people’s access to resources, but the neighborhood characteristics that affect the availability of resources are not included in this analysis. Therefore, multilevel models with more detailed neighborhood features are needed in future related studies.

## Conclusions

This study analyzes the income-health relationship and how it is mediated through people’s social networking time based on a national-level representative sample from the GSS. The structure and nature of people’s social networks are associated with their structural embeddedness and contextual characteristics. The test of the mediation effect of social networking time on the relationship between income and health shows that neighborhood socializing may influence people’s mental health as a result of the sense of relative deprivation and the obligatory reciprocity entailed in neighborhood ties. This finding confirms the unequal distribution of HRQoL among people with different income levels and verifies its reproduction through neighborhood social ties.

## Appendix

### Bootstrap check of multiple linear regressions

With respect to the non-normal distribution of social networking time with neighbors and number of days with various health conditions, the significance of multiple linear regressions was checked using bootstrap methods with no distribution assumptions (Tables 5 and 6).

**Table 5:** Bootstrap check of income and social networking time

Variables	Model 11	Model 12
Income	−0.133^***^	−0.137^***^
	(2.141)	(2.295)
Age	− 0.621^***^	− 0.603^***^
	(0.842)	(0.859)
Age^2^	0.534^***^	0.528^***^
	(0.009)	(0.009)
Sex (male)	0.063^***^	0.063^***^
	(3.277)	(3.234)
Race (white)	0.007	0.003
	(3.894)	(4.108)
Marital status (married)	−0.087^***^	−0.080^***^
	(3.657)	(3.499)
Working status (working)	0.024	0.024
	(9.112)	(9.324)
Place of living (urban)	0.009	0.008
	(4.789)	(5.107)
Geographical mobility (same city since age 16)		0.003
		(3.466)
Number of children		−0.032
		(1.237)
Year fixed effects	yes	yes
*N*	3037	3029
*R* ^2^	0.070	0.071

Standardized beta coefficients; standard errors in parentheses

^*^ *p* < 0.05, ^**^ *p* < 0.01, ^***^ *p* < 0.001

**Table 6:** Bootstrap check of income, social networking time and HRQoL

Variables	Days of poor mental health		Days of poor physical health		Days of activity limitations		General assessment of health	
	Model 13	Model 14	Model 15	Model 16	Model 17	Model 18	Model 19	Model 20
Income	− 0.127^***^	−0.118^***^	− 0.106^***^	−0.102^***^	− 0.069^*^	−0.065^*^	0.219^***^	0.223^***^
	(0.164)	(0.165)	(0.139)	(0.152)	(0.112)	(0.109)	(0.022)	(0.023)
Place of living (urban)	0.002	0.002	0.016	0.016	0.012	0.012	0.049^**^	0.049^**^
	(0.389)	(0.392)	(0.334)	(0.352)	(0.233)	(0.226)	(0.057)	(0.060)
Age	0.082	0.124	−0.010	0.013	−0.043	−0.026	−0.443^***^	−0.424^***^
	(0.061)	(0.061)	(0.061)	(0.057)	(0.036)	(0.036)	(0.009)	(0.009)
Age^2^	−0.141	−0.177	0.066	0.046	0.064	0.050	0.354^**^	0.338^**^
	(0.001)	(0.001)	(0.001)	(0.001)	(0.0004)	(0.0004)	(0.0001)	(0.0001)
Sex (male)	−0.054^**^	−0.058^***^	−0.026	− 0.028	−0.018	− 0.020	−0.012	− 0.014
	(0.241)	(0.239)	(0.234)	(0.218)	(0.153)	(0.142)	(0.036)	(0.035)
Race (white)	0.066^***^	0.065^***^	0.032	0.032	0.031	0.031	0.024	0.024
	(0.287)	(0.297)	(0.244)	(0.260)	(0.154)	(0.153)	(0.044)	(0.044)
Marital status (married)	−0.027	−0.021	0.005	0.008	−0.024	−0.022	− 0.031	−0.029
	(0.283)	(0.271)	(0.232)	(0.237)	(0.160)	(0.164)	(0.041)	(0.041)
Working status (working)	−0.116^***^	−0.117^***^	− 0.207^***^	−0.208^***^	− 0.285^***^	−0.285^***^	0.088^***^	0.087^***^
	(1.158)	(1.244)	(1.413)	(1.412)	(1.346)	(1.304)	(0.128)	(0.121)
Social networking time		0.067^**^		0.037		0.026		0.030
		(0.002)		(0.001)		(0.001)		(0.002)
Year fixed effects	yes	yes	yes	yes	yes	yes	yes	yes
*N*	3037	3037	3030	3030	3032	3032	3036	3036
*R* ^2^	0.045	0.049	0.059	0.061	0.092	0.093	0.063	0.064

Standardized beta coefficients; standard errors in parentheses

^*^ *p* < 0.05, ^**^ *p* < 0.01, ^***^ *p* < 0.001

There is no change in the *p* value of income in the bootstrap results between Table 5 and Table 3 in the relationship between income and social networking time. In Table 6, the association between social networking time and mental health is significant at the 99% level in Model 14. The effect of social networking time on physical health is not significant at the 95% level in Model 16. The significance level of the relationship between income and days of activity limitations changes from 99.9% in Model 7 and 99% in Model 8 to 95% in Models 17 and 18.

## Abbreviations

CDC: Centers for Disease Control and Prevention
GSS: General Social Survey
HRQoL: Health-related quality of life

## References

[1] Mackenbach JP, Stirbu I, Roskam AJR, Schaap MM, Menvielle G, Leinsalu M, Kunst AE (2008). Socioeconomic inequalities in health in 22 European countries. N Engl J Med.

[2] Eikemo TA, Huisman M, Bambra C, Kunst AE (2008). Health inequalities according to educational level in different welfare regimes: a comparison of 23 European countries. Sociol Health Illn.

[3] Bor J, Cohen GH, Galea S (2017). Population health in an era of rising income inequality: USA, 1980–2015. Lancet.

[4] Hu Y, van Lenthe FJ, Borsboom GJ (2016). Trends in socioeconomic inequalities in self-assessed health in 17 European countries between 1990 and 2010. J Epidemiol Community Health.

[5] Lubetkin EI, Jia H, Franks P (2005). Relationship among sociodemographic factors, clinical conditions, and health-related quality of life: examining the EQ-5D in the US general population. Qual Life Res.

[6] Strand BH, Grøholt EK, Steingrímsdóttir ÓA, Blakely T, Graff-Iversen S, Næss Ø (2010). Educational inequalities in mortality over four decades in Norway: prospective study of middle aged men and women followed for cause specific mortality, 1960-2000. BMJ..

[7] Cutler DM, Lange F, Meara E, Richards-Shubik S, Ruhm CJ (2011). Rising educational gradients in mortality: the role of behavioral risk factors. J Health Econ.

[8] Centers for Disease Control and Prevention. Strategies for reducing health disparities-selected CDC-sponsored interventions. United States MMWR. 2016;65(1):1–69.

[9] Marmot M (2002). The influence of income on health: views of an epidemiologist. Health Aff.

[10] Luo J, Zhang X, Jin C, Wang D (2009). Inequality of access to health care among the urban elderly in northwestern China. Health Policy.

[11] Casey PH, Szeto K, Lensing S, Bogle M, Weber J (2001). Children in food-insufficient, low-income families: prevalence, health, and nutrition status. Arch Pediatr Adolesc Med.

[12] Heritage Z, Wilkinson RG, Grimaud O, Pickett KE (2008). Impact of social ties on self reported health in France: is everyone affected equally?. BMC Public Health.

[13] Wilkinson RG (1996). Unhealthy societies – the afflictions of inequality.

[14] Eibner CE, Sturm R, Gresenz CR (2004). Does relative deprivation predict the need for mental health services?. J Ment Health Policy Econ.

[15] Hawkley LC, Hughes ME, Waite LJ, Masi CM, Thisted RA, Cacioppo JT (2008). From social structural factors to perceptions of relationship quality and loneliness: the Chicago health, aging, and social relations study. J Gerontol Ser B Psychol Sci Soc Sci.

[16] Ashida S, Heaney CA (2008). Differential associations of social support and social connectedness with structural features of social networks and the health status of older adults. J Aging Health.

[17] Rosenquist JN, Fowler JH, Christakis NA (2011). Social network determinants of depression. Mol Psychiatry.

[18] Onnela JP, Christakis NA (2012). Spreading paths in partially observed social networks. Phys Rev E.

[19] Smith KP, Christakis NA (2008). Social networks and health. Annu Rev Sociol.

[20] Caughy MOB, O’Campo PJ, Muntaner C (2003). When being alone might be better: neighborhood poverty, social capital, and child mental health. Soc Sci Med.

[21] Bianchi EC, Vohs KD (2016). Social class and social worlds: income predicts the frequency and nature of social contact. Soc Psychol Personal Sci.

[22] Menati W, Baghbanian A, Asadi-Lari M, Moazen J, Menati R, Sohrabivafa M (2017). Health-related quality of life and socioeconomic status: inequalities among adults in west of Iran. Iran Red Crescent Med J.

[23] Rajmil L, Herdman M, Ravens-Sieberer U, Erhart M, Alonso J (2014). Socioeconomic inequalities in mental health and health-related quality of life (HRQOL) in children and adolescents from 11 European countries. Int J Public Health.

[24] Tan Z, Shi F, Zhang H, Li N, Xu Y, Liang Y (2018). Household income, income inequality, and health-related quality of life measured by the EQ-5D in Shaanxi, China: a cross-sectional study. Int J Equity Health.

[25] Centers for Disease Control and Prevention (2017). Health-related quality of life.

[26] Kaplan RM, Bush JW (1982). Health-related quality of life measurement for evaluation research and policy analysis. Health Psychol.

[27] Smith TW, Marsden P, Hout M, et al. General Social Surveys, 2002, 2006, 2008, & 2014 [machine-readable data file]. https://gssdataexplorer.norc.org.

[28] Smith TW (2016). The general social surveys: GSS project report no. 32.

[29] Centers for Disease Control and Prevention (2018). How Does CDC Measure Population Health-Related Quality of Life?.

[30] Andresen EM, Fouts BS, Romeis JC (1999). Performance of health-related quality-of-life instruments in a spinal cord injured population. Arch Phys Med Rehabil.

[31] Newschaffer CJ (1998). Validation of the BRFSS HRQoL measures in a statewide sample.

[32] Andresen EM, Catlin TK, Wyrwich KW (2003). Retest reliability of surveillance questions on health related quality of life. J Epidemiol Community Health.

[33] Ligon EGSS (1994). Methodological Report No. 64: The development and use of a consistent income measure for the General Social Survey.

[34] Glanville JL, Andersson MA, Paxton P (2013). Do social connections create trust? An examination using new longitudinal data. Social Forces.

[35] Putnam RD (2000). Bowling alone: the collapse and revival of American community.

[36] Baron RM, Kenny DA (1986). The moderator–mediator variable distinction in social psychological research: conceptual, strategic, and statistical considerations. J Pers Soc Psychol.

[37] Haren MT (2012). Abdominal adiposity and obstructive airway disease: testing insulin resistance and sleep disordered breathing mechanisms. BMC Pulm Med.

[38] Lumley T, Diehr P, Emerson S (2002). The importance of the normality assumption in large public health data sets. Annu Rev Public Health.

[39] Doran T, Fullwood C, Kontopantelis E (2008). Effect of financial incentives on inequalities in the delivery of primary clinical care in England: analysis of clinical activity indicators for the quality and outcomes framework. Lancet.

[40] Elo IT (2009). Social class differentials in health and mortality: patterns and explanations in comparative perspective. Annu Rev Sociol.

[41] Johnson W, Krueger RF (2006). How money buys happiness: genetic and environmental processes linking finances and life satisfaction. J Pers Soc Psychol.

[42] Lachman ME, Weaver SL (1998). The sense of control as a moderator of social class differences in health and well-being. J Pers Soc Psychol.

[43] Kraus MW, Keltner D (2009). Signs of socioeconomic status a thin-slicing approach. Psychol Sci.

[44] Ziersch AM, Baum FE, MacDougall C (2005). Neighborhood life and social capital: the implications for health. Soc Sci Med.

[45] Kawachi I, Berkman LF (2001). Social ties and mental health. J Urban Health.

[46] Stafford M, Marmot M (2003). Neighborhood deprivation and health: does it affect us all equally?. Int J Epidemiol.

[47] Stafford M, De Silva M, Stansfeld S (2008). Neighborhood social capital and common mental disorder: testing the link in a general population sample. Health Place.

[48] Belle DE (1983). The impact of poverty on social networks and supports. Marriage Fam Rev.

[49] Liem R, Liem J (1978). Social class and mental illness reconsidered: the role of economic stress and social support. J Health Soc Behav.

[50] Durkheim E (1970). Suicide: a study in sociology. (translated by John a. Spalding and George Simpson).

